# Prof. Peaslee’s Synopsis of Lectures on General and Human Physiology. 1848

**Published:** 1848-05

**Authors:** 


*Prof. Peaslee's Synopsis of Lectures on General and Human Physiology.* 1848.

This synopsis, for which we are indebted to the polite attention of the author, presents a comprehensive digest of all the more important points embraced in general and human physiology. While it shows the author's course to be thorough and complete, and must be of material service to those in attendance thereupon, it is calculated to serve an useful purpose for the practitioner who may wish to review the subjects, in order to refresh his recollection of the multitude of facts which they embrace.

We have also been favored with an introductory lecture by Prof. P., which we have perused with much gratification.

